# *Wolbachia* Do Not Induce Reactive Oxygen Species-Dependent Immune Pathway Activation in *Aedes albopictus*

**DOI:** 10.3390/v7082836

**Published:** 2015-08-13

**Authors:** Jennifer C. Molloy, Steven P. Sinkins

**Affiliations:** 1 Peter Medawar Building for Pathogen Research and Department of Zoology, University of Oxford, Oxford OX1 3SY, UK; E-Mail: jenny.molloy@cantab.net; 2 Biomedical and Life Sciences, Lancaster University, Lancaster LA1 4YQ, UK

**Keywords:** mosquito, Aedes, arbovirus, dengue, chikungunya, ROS

## Abstract

*Aedes albopictus* is a major vector of dengue (DENV) and chikungunya (CHIKV) viruses, causing millions of infections annually. It naturally carries, at high frequency, the intracellular inherited bacterial endosymbiont *Wolbachia* strains *w*AlbA and *w*AlbB; transinfection with the higher-density *Wolbachia* strain *w*Mel from *Drosophila melanogaster* led to transmission blocking of both arboviruses. The hypothesis that reactive oxygen species (ROS)-induced immune activation plays a role in arbovirus inhibition in this species was examined. In contrast to previous observations in *Ae. aegypti*, elevation of ROS levels was not observed in either cell lines or mosquito lines carrying the wild-type *Wolbachia* or higher-density *Drosophila* *Wolbachia* strains. There was also no upregulation of genes controlling innate immune pathways or with antioxidant/ROS-producing functions. These data suggest that ROS-mediated immune activation is not an important component of the viral transmission-blocking phenotype in this species.

## 1. Introduction

*Wolbachia* are maternally transmitted, intracellular bacterial endosymbionts that can spread to high frequency in insect populations by manipulating their reproduction, in particular using patterns of sterility known as cytoplasmic incompatibility to give a reproductive advantage to *Wolbachia*-infected females [1]. Some *Wolbachia* transinfections in *Aedes* mosquitoes, especially with the *Drosophila*-derived *w*MelPop and *w*Mel strains, have been found to block the transmission of human arboviruses such as dengue [2,3,4,5,6,7]. The *w*Mel strain has been stably introduced into wild populations of *Aedes aegypti* [8,9], and expanded trials of the use of *Wolbachia* for arbovirus control are planned or underway. In this context it is important to gain a better understanding of the mechanisms by which the bacterium can inhibit virus transmission.

In *Wolbachia*-transinfected *Ae. aegypti*, chronic upregulation of insect innate immune pathways, including antimicrobial peptides (AMPs) and other immune effectors has been observed and may play a role in the inhibition of arboviruses [2,3,10,11]. In a *w*AlbB-transinfected line, increased ROS production was also reported [11] and this was linked to the activation of the Toll innate immune pathway, which has previously been show to control DENV infection [12,13]; it was hypothesised that this activation was the major causal factor in DENV inhibition. Upregulation of antioxidant genes was also observed [11]. The link between ROS and Toll induction was supported by provision of H_2_O_2_-supplemented sugar water to *Wolbachia*-uninfected *Ae.* *aegypti*, whereupon Toll pathway genes were upregulated. Conversely, Toll genes were downregulated following knockdown of ROS-generating enzymes via RNAi [11]. Under this model, DENV inhibition is proposed to be mediated by Toll-induced AMPs such as cecropins and defensins.

Innate immune pathway signalling molecules and effectors are distinct proteins, but some small molecules such as ROS moieties play a dual role. They are implicated in both immune signalling and direct killing of pathogens in a number of systems [14]. In both vertebrate macrophages and insect haemocytes, oxidative bursts transiently increase ROS levels in the vicinity of phagocytosed microbes and are believed to directly oxidise microbial targets and inflict DNA damage in conjunction with reactive nitrogen species (RNS). In mosquitoes there is evidence that ROS modulate immunity against *Plasmodium* and bacteria. For example, *An. gambiae* lines with naturally higher systemic H_2_O_2_ levels show higher antibacterial activity which is reduced upon antioxidant treatment [15] and exogenous ROS from gut microflora can induce *Plasmodium* resistance [16]. In terms of indirect antimicrobial action, there is evidence from *An. gambiae* immunity to *Plasmodium* that dual oxidase (Duox) generated ROS may be involved in peroxidase mediated cross-linking of protective midgut layers [17] and melanotic encapsulation [18], while NADPH oxidase 5 (NOX5) generated ROS mediate the TEP-1 complement response [19]. Furthermore, ROS could activate the mitogen-activated protein kinase (MAPK) and Phosphatidylinositol 3-kinase/Akt (PI3K/AKT) signalling pathways [20], which are thought to modulate innate immune responses. ROS activation of NF-κB pathways occurs in mammals [21] and there is ROS-mediated cross-talk between Jnk and NF-κB [22].

ROS can be beneficial for hosts due to their immune role, but they can also induce oxidative stress by directly damaging DNA and interfering with signalling and metabolic pathways, reducing longevity and fecundity [23,24]. Maintenance of redox homeostasis is also important for controlling mosquito gut microbiota, which proliferate following blood feeding due to ingestion of the antioxidant heme [25]. There is a balance to be struck by the host in controlling ROS generation and antioxidant production to target pathogens while minimising systemic damage.

In the invasive dengue and chikungunya virus vector *Aedes albopictus,* which naturally carries the *w*AlbA and *w*AlbB strains of *Wolbachia* at high frequency, no upregulation of a selection of AMPs was observed in wildtypes or a *w*Mel transinfection, despite complete blocking of DENV transmission in the latter [5]. Viral inhibition did positively correlate with *Wolbachia* density [5], as also shown for in cell lines [26,27]. However, increased ROS levels were previously reported by flow cytometry and microscopy in naturally infected versus antibiotic-cured *Ae. albopictus* cell lines; upregulation of antioxidant genes was also observed [28]. The aim of this study was to reconcile these differing observations in *Ae. albopictus*, and relate them to work in *Ae. aegypti*, in order to better understand whether ROS-mediated immune activation may be an important component of arboviral inhibition in this system. Direct biochemical measurement of ROS was undertaken, and ROS-modulating and immune pathway regulator gene transcription examined, in both cell lines and mosquito lines carrying wild-type and transinfected *Wolbachia* strains.

## 2. Materials and Methods

### 2.1. Mosquito and Cell Lines

All mosquito lines were reared at 27 °C and 70% relative humidity as previously described [5]. The tetracycline cured Aa23 cell line Aa23-T [29,30] was transinfected with *w*MelPop from *D. melanogaster* *w^1118^* and *w*Mel from *D. melanogaster* yw^67c23^ as previously described [30,31]. Cells were grown in 25 cm^2^ or 75 cm^2^ flasks kept at 27 °C in Schneiders Medium (Promo-Cell, Heidelberg, Germany) with 10% fetal bovine serum (FBS) and 1% penicillin-strepticillin (pen-strep, Gibco-Life Technologies, Paisley, UK). Standard passage time was 5–7 days. All cell line assays were conducted at 70%–80% confluency.

### 2.2. Gene Transcription qRT-PCR Assays

RNA was extracted from pools of three 7-day old adult females or from cultures of Aa23 in 12-well plates, seeded from 250 k cells and incubated for 72 h. Samples were homogenised in 100 μL Trizol (Invitrogen-Life technologies, Paisley, UK) or Tri Reagent (Sigma-Aldrich, Gillingham, UK) per mosquito or 10^6^ Aa23 cells using an electric motor driven pestle (Kimble Chase, Vineland, NJ, USA), or by grinding with borosilicate beads at 40 Hz for 4 min using a Qiagen TissueLyser LT (Qiagen, Manchester, UK). RNA was extracted per manufacturer's instructions and treated with DNase I (NEB) then purified by phenol-chloroform extraction. Samples were quantified and quality checked using Nanodrop analysis and those with high purity (A260:230 >1.90 and A260:280 ratios of >2.00) were used to synthesise cDNA from 1 μg RNA using the High Capacity RNA-to-cDNA Kit (Applied Biosystems-Life Technologies, Paisley, UK) as per manufacturer’s instructions.

The *Ae.* *albopictus* transcriptome database albopictusexpression.org [32] was interrogated using BLAST to identify transcripts of immune pathway and ROS-related genes, and qRT-PCR primers were designed as per Table 1. All primers were tested using a template standard curve and achieved efficiencies of 1.8–2.1 (80%–110%). Standard curves were run on all plates to assess efficiency per run. Quantitative PCR was performed in a MJ Research PTC-200 DNA Engine using a Chromo4 detection system and Opticon Monitor Software v3.1 (all manufactured by Bio-Rad Laboratories, Hemel Hempstead, UK). SYBR Green EXPRESS qPCR Supermix Universal (Life Technologies, Paisley, UK) and the iQ SYBR Green supermix (Bio-Rad Laboratories) were used in 10 μL reaction as per manufacturers’ instructions. Cycling conditions were 4 min at 95 °C followed by 40 cycles of 95 °C for 15 s and 59 °C for 30 s. Some independent repeats used SYBR Green JumpStart Taq ReadyMix (Sigma-Aldrich) on a Stratagene Mx3005P Real-Time PCR System (Agilent Technologies, Stockport, UK) using the same mix and thermocycler settings.

**Table 1:** Oligonucleotide primers for qRT-PCR of immune pathway and ROS-related genes in *Ae. albopictus*. Transcript numbers refer to albopictusexpression.org Aalb-OocyteEmbryoPharatelarvaeAssembly- identifiers [32] from which the primers were designed.

Gene	Forward Primer	Reverse Primer	Product	Transcript
CAC	TGTTCAGCTCGTCTTCGTCA	GGACTGGTGGTACTGGTGCT	71bp	140339
Rel1	GCTATCGGACACTGGACGTT	TGCGTTTCTTTCGTTTGGCT	132bp	145853
CSP	AGAATGCGTAGCGGAGTGTC	GACCGGTGAGAACATAACGAA	137bp	082813
Rel2	CCGCATCTTCATTCAGCTTT	TTTCGATACCAATCGGAGATG	81bp	008087
PIAS	TCAAACCGGCAGATTACACA	CGGGAAGGTCTTCTTGCTTT	57bp	135921
STAT	ATCACCTGCCCTATCAACCG	ACGACGACGCAAACATATCG	66bp	146249
TAK1	GTTCGGAAAATCCCAACTCAGG	CTCCGCAACAAACCATGGAAG	62bp	099738
Duox	GGGAATAGCAAGGCTTCCGT	ACCAAGCGTTTGTGATTGGC	71bp	144190
DuoxA	TCAGCAATGGTGGGAACCTC	ACAAAGTCAAAGCGCAGCAG	115bp	102149
OXR1	CTACTCGTGGTCCCTGGTGT	ATTGGGCTTTCCAGTTTGTG	90bp	078559
Gpx	CCAACGAGGAGATCAAGCAC	TCAAACTTGGCTCCCTTCTG	52bp	063197
CuZnSOD	ATGTCAAGGGCACCATCTTC	GGCTTGAGTCCAGTCACCTC	82bp	143681

Ct values were analysed using the R package MCMC.qPCR, which employs a Bayesian approach to data analysis. An informed model was fitted assuming no variation in the control gene S17 (primers AS17-F AAGCCCCTGCGTAACAAGAT, AS17-R GTTATCTCTGCGCTCACGTTC) and log(2) fold-changes were derived along with Bayesian 95% credible intervals and pMCMC values. Primer efficiencies were calculated per experiment using a standard cDNA curve and fed into the informed model.

### 2.3. Wolbachia Density qPCR Assay

A universal Culicidae *Wolbachia* density assay was designed, allowing absolute qPCR quantification of host and *Wolbachia* genome copy number based on a plasmid standard curve. A single copy nucleotide sequence conserved across mosquito species was identified: a 93 bp conserved regulatory element upstream of the homothorax (HTH) gene [33]. Primers were designed using PrimerBLAST to amplify this region across all sequenced Culicidae species and *Wolbachia* surface protein (Wsp) sequences in the insect-infecting *Wolbachia* strains for which genomes were available: Mos qHTH F2 TGGTCCTATATTGGCGAGCTA; Mos qHTH RTCGTTTTTGCAAGAAGGTCA; qWSPallF3-ATCTTTTATAGCTGGTGGTGGT [2], qWSPallR1-AAAGTCCCTCAACATCAACCC.

DNA was extracted from pools of three 7-day old adult females or from confluent cultures of Aa23 cell lines in 12-well plates. Samples were homogenised in 100 μL cetyltrimethyl ammonium bromide (CTAB) buffer per mosquito (0.1 M Tris HCl pH 8.0, 1 M NaCl, 10 mM EDTA, 1% CTAB). Following incubation at 95 °C for 30 min, the sample was left to cool and an equal volume of chloroform was added prior to centrifugation at 15,000× *g* for 25 min at 4 °C. The aqueous layer was transferred to a new tube and DNA was precipitated with an equal volume of isopropanol then washed twice with 70% ethanol prior to resuspension in TE buffer and quantification using a Nanodrop spectrophotometer. DNA was diluted to 50 ng/μL and 100 ng was added to a 10 μL qPCR reaction using SYBR Green EXPRESS qPCR Supermix Universal (Life Technologies) as per manufacturers’ instructions.

*Wolbachia* genomes per host genome were determined from a linear model fitted to the known plasmid standard curve values. This data was tested for normality and homogeneity of variances using the Shapiro and Bartlett tests respectively. Normal data was then assessed using a two-way analysis of variance (ANOVA) followed by Tukey Honestly Significant Differences (Tukey HSD) *post-hoc* pairwise testing. Data which deviated from the normal distribution was analysed via a Kruskal-Wallis test using a Bonferroni adjustment for multiple comparisons.

### 2.4. Hydrogen Peroxide Assays

Hydrogen Peroxide H_2_O_2_ is the most stable form of ROS and thus acts as a correlate of overall ROS levels, it also has a highly specific biochemical assay which overcomes some of the issues in interpreting data inherent in other ROS probes, some of which have been shown to be highly non-specific [34]. H_2_O_2_ generation by live cells was assessed using an Amplex Red Hydrogen Peroxide Kit (Invitrogen). Cells in the exponential growth phase (four days post passage in 75 cm^2^ flasks) were harvested in fresh Schneiders solution (+10% FBS, +1% penicillin-streptomycin) and viability was assessed by Trypan blue staining to ensure all lines had similar viability. 150,000 cells were plated per well of a flat bottomed 96-well microplate and allowed to settle for one hour in the dark at 25 °C. Media was then removed, cells were washed once with Phosphate-buffered saline (PBS) and 50 μL Amplex Buffer in PBS was added followed by 50 μL Amplex reaction solution (as per manufacturers’ guidelines) immediately prior to insertion into a FLUOstar microplate reader (BMG LabTech, Aylesbury, UK).

Pools of three mosquitoes were collected on ice in 2 mL tubes containing 1000 μL PBS with 2 mg/mL 3-Amino-1,2,4-triazole (3-AT) and three borosilicate beads. Samples were homogenised at 50 Hz for 5 min in a TissueLyser LT (Qiagen) and centrifuged at 20,000× *g* for 15 min at 4 °C. The supernatant was transferred to a new tube and centrifuged again at 20,000× *g* for 10 min at 4 °C before 10 μL supernatant was added to a 96 well flat bottomed plate for the protein assay and 250 μL was added to a 10,000 k molecular weight cut-off (MWCO) Spinfilter Ultrafiltration centrifugal filter (VWR) and centrifuged at 14,000× *g* for 20 min. 50 μL of filtrate was added to a separate flat bottomed 96-well plate for the H_2_O_2_ assay. Both plates were stored on ice until assays began.

200 μL Bradford Reagent (Sigma-Aldrich) was added to samples in the protein assay plate, which was then shaken at 100 rpm for 30 s and incubated at 25 °C in the dark for 10 min before being read at 595 nm using a FLUOstar microplate reader (BMG LabTech).

The Amplex Red H_2_O_2_ Assay (Invitrogen) was used according to manufacturers’ instructions with 50 μL Amplex reagent (containing 2× Amplex buffer) added to the 50 μL sample prepared above. The plate was then incubated in the FLUOstar microplate reader at 25 °C where it was read kinetically at 545/590 nm excitation/emission over 40 cycles of 60 s with shaking at 100 rpm every two cycles to reduce clumping. The 30 min reading was used after confirmation that the assay kinetics were linear at this time point. Readings from protein and H_2_O_2_ samples were compared to standard curves of known concentration of albumin (Sigma Aldrich) and H_2_O_2_ (Invitrogen) in the respective assays. H_2_O_2_ was then normalised to protein content for adult assays and cell count for cell line assays (verified by post assay counts from randomly selected wells within each line). This data was tested for normality and homogeneity of variances using the Shapiro and Bartlett tests respectively then assessed using a two-way ANOVA followed by Tukey HSD pairwise testing or by Kruskal-Wallis tests.

## 3. Results

### 3.1. Innate Immune Pathway Gene Transcription

**Figure 1:**
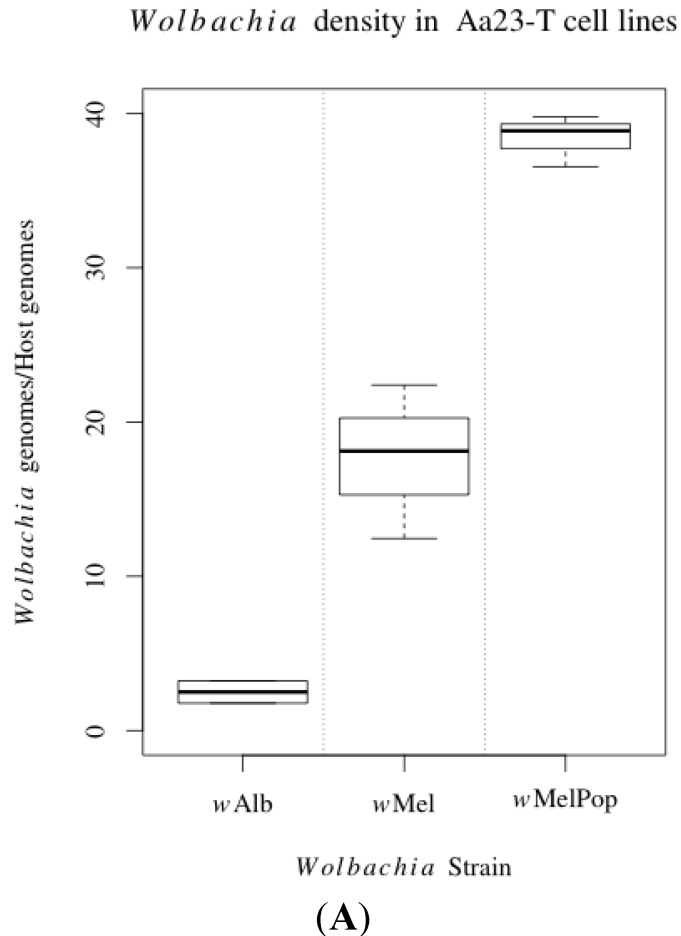

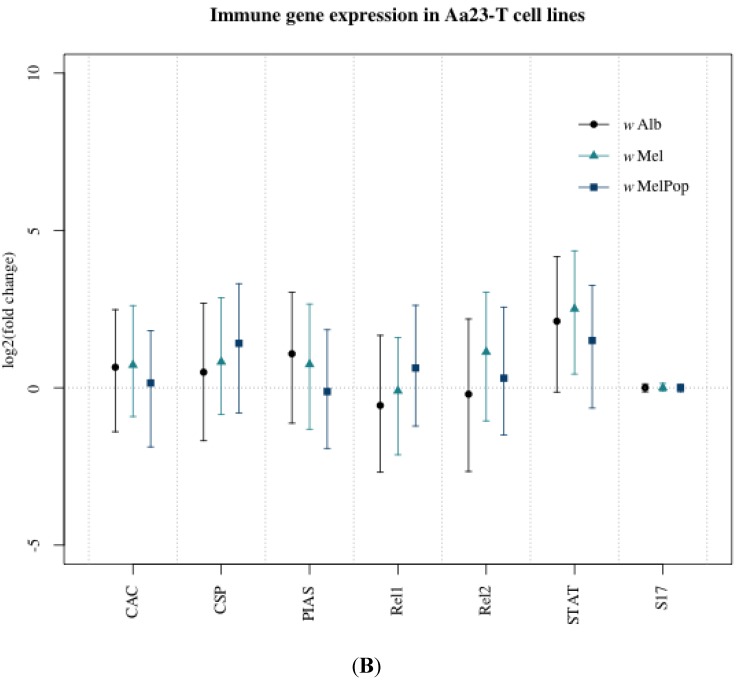
(**A**) Ratio of *Wolbachia* genome copies to *Ae. albopictus* genome copies detected in Aa23-T.*w*AlbB, Aa23-T.*w*Mel and Aa23-T.*w*MelPop cell lines. Genome copies were determined by absolute quantification using a standard curve of plasmid DNA containing the genes of interest. Results are from two or three biological replicates; (**B**) Immune pathway gene transcription in *Ae. albopictus* Aa23-T.*w*AlbB, Aa23-T.*w*Mel and Aa23-T.*w*MelPop cell lines compared to Aa23-T, normalised to the housekeeping gene S17. Points represent the mean of three biological replicates and error bars denote 95% confidence intervals calculated using Bayesian MCMC methods in MCMC.qPCR R package.

The transcription of immune pathway regulator genes CAC, Rel1, CSP, Rel2, PIAS and STAT was assessed in the *Wolbachia*-uninfected *Ae. albopictus* cell line Aa23-T compared to the same cell line infected with *w*Alb, *w*Mel and *w*MelPop using qRT-PCR. These transinfected lines differed significantly in density, respectively harbouring a mean of 2.5, 17.7 and 38.4 *Wolbachia* genomes per host genome (Figure 1A; one-way ANOVA, F = 71.00, *p* < 0.001. Tukey HSD, *p* < 0.01 for all comparisons). Only one significant difference was found, a 5.66-fold increase in STAT expression in Aa23-T.wMel (pMCMC = 0.014) (Figure 1B). This was not supported in the most densely infected line Aa23-T.wMelPop, which showed a non-significant STAT increase of 2.66-fold. There was no increase in expression of the Toll pathway transcription factor Rel1 or decrease in expression of its negative regulator CAC, both of which might be expected during Toll activation.

**Figure 2:**
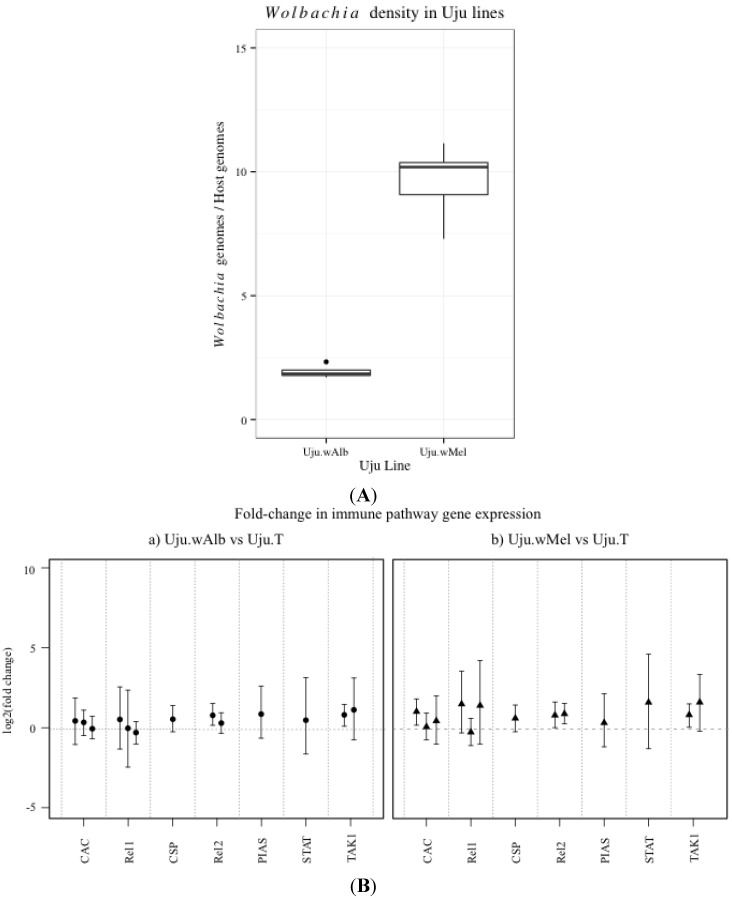
(**A**) Ratio of *Wolbachia* genome copies to *Ae. albopictus* genome copies detected in Uju.*w*Alb and Uju.*w*Mel adult lines. Genome copies were determined by absolute quantification using a standard curve of plasmid DNA containing the genes of interest. Results are from 4–5 biological replicates of pools containing three females; (**B**) Immune gene transcription in *Ae. albopictus* lines Uju.*w*Alb and Uju.*w*Mel compared to Uju.T. Points represent means of 3–5 biological replicates and error bars denote Bayesian 95% credible intervals. Each bar within a gene category represents an independent experiment.

Immune gene transcription in the *Ae. albopictus* mosquito lines Uju.*w*Alb and Uju.*w*Mel was compared to uninfected Uju.T, normalised to the housekeeping gene S17. Uju.*w*Alb had a mean *Wolbachia* infection density of 1.93 *Wolbachia* genomes per host genome, while Uju.*w*Mel had a substantially higher infection density of 9.62 (Figure 2). No consistent significant difference in CAC, Rel1 or Rel2 or TAK1 was observed.

### 3.2. H_2_O_2_ Levels in Ae. albopictus Adults and Cell Lines

**Figure 3:**
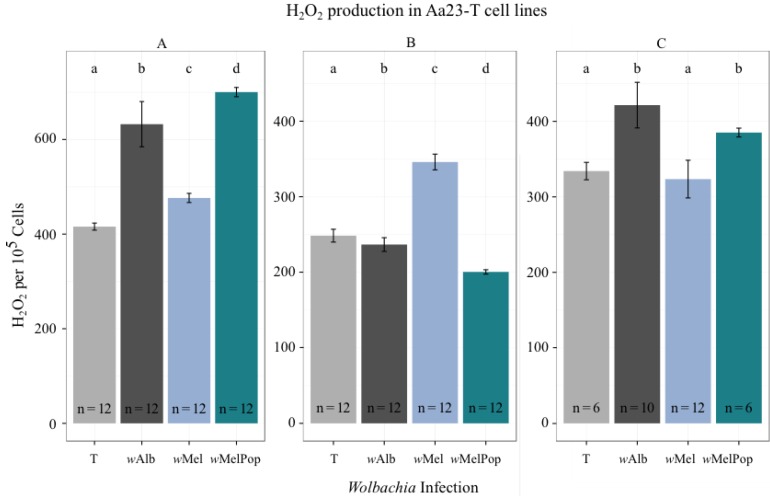
H_2_O_2_ detected in Aa23-T cells, normalised to cell count. Graphs A, B and C represent independent experiments. Bars represent mean of n samples as shown and error bars represent the standard deviation of the mean. Lower case letters represent significant pairwise differences at *p* <0.05 derived from Kruskal-Wallis tests.

**Figure 4:**
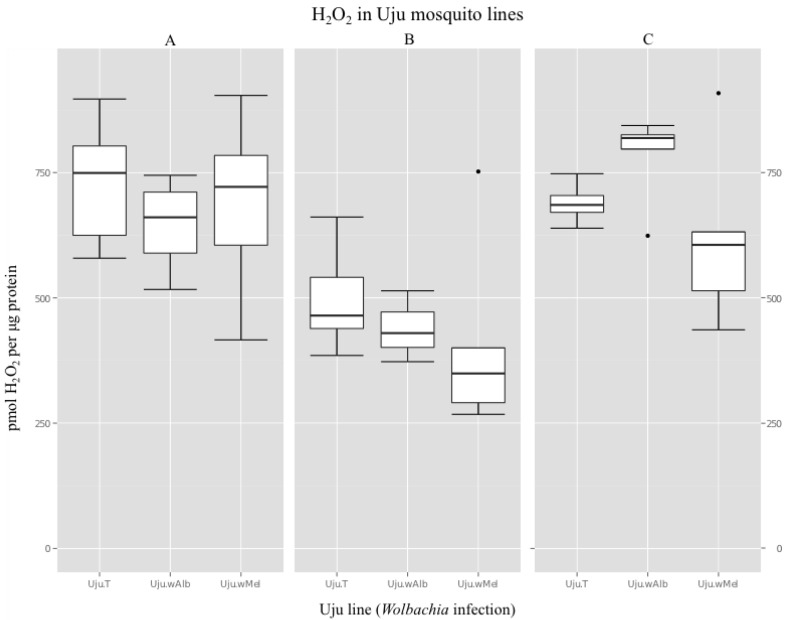
H_2_O_2_ detected in adult homogenates of *Ae. albopictus* lines Uju.T, Uju.*w*Alb and Uju.*w*Mel, normalised to sample protein content. Data was obtained from six pools of three adult females per line in each of three independent experiments.

All Uju mosquito and Aa23-T cell lines were assayed for H_2_O_2_ to determine if *Wolbachia* infection increased ROS production and if any effect correlated with *Wolbachia* density (Figure 3). Relative H_2_O_2_ generation in Aa23-T cells across three independent experiments was significantly different between lines as analysed by Kruskal-Wallis testing for non-normal data (A: χ^2^ = 43.80, df = 3, *p*-value = 1.67e−09. B: χ^2^= 41.89, df = 3, *p*-value = 4.23e−09. C: χ^2^= 26.04, df = 3, *p*-value = 9.36e−06). *Post-hoc* bootstrap ratio testing showed no consistent significant differences between uninfected and infected lines as denoted on Figure 4, with each infected line producing slightly more H_2_O_2_ than uninfected in two out of three experiments but slightly less H_2_O_2_ in the third. No overall trend towards increased H_2_O_2_ in *Wolbachia*-infected cell lines was thus observed.

In crude adult extracts, there was no trend towards increased H_2_O_2_ levels in *Wolbachia*-infected lines. A typical result was a mean of 730 pmol H_2_O_2_ per μg protein in the uninfected Uju.T line compared to 646 and 689 pmol respectively in Uju.*w*Alb and Uju.*w*Mel, resulting in no significant difference between the lines (Figure 4a, ANOVA, F = 0.592, *p* = 0.566). Two further independent experiments had means in the same approximate range (min = 619 pmol, max = 1154 pmol) and showed no difference between lines (Figure 4b, ANOVA, F = 1.06, *p* = 0.371. Figure 4c, ANOVA, F = 1.548, *p* = 0.252).

### 3.3. ROS-Generating Enzyme and Antioxidant Gene Transcription

The lack of a detectable difference in H_2_O_2_ between lines could be because ROS production is similar or alternatively because there is a compensating increase in antioxidant enzyme expression to maintain redox homeostasis [28]. To differentiate between these hypotheses, transcription of a selection of antioxidant and ROS-generating genes were assessed by qRT-PCR.

**Figure 5:**
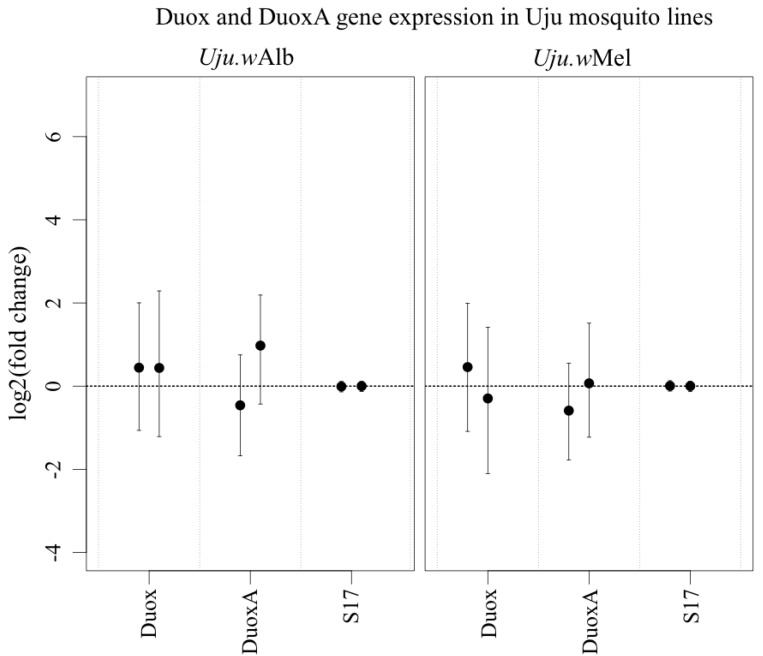
Transcription of Duox and DuoxA in Uju.*w*Alb and Uju.*w*Mel adult females, normalised to S17 transcription and to uninfected Uju.T. Points represent mean of 3–5 pools of three females and error bars denote Bayesian 95% credible intervals. Each bar within a gene category represents an independent experiment.

**Figure 6:**
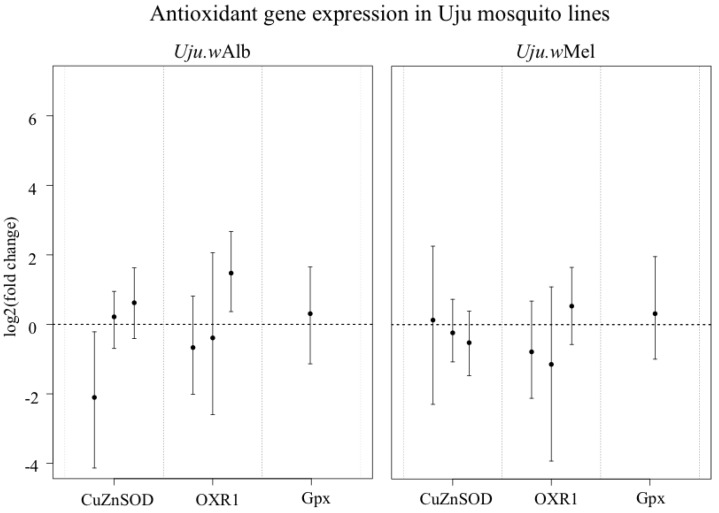
Transcription of CuZnSOD, Gpx and OXR1 in Uju.*w*Alb and Uju.*w*Mel adult females, normalised to S17 transcription and to uninfected Uju.T. Points represent mean of 3–5 pools of three females and error bars denote Bayesian 95% credible intervals. Each bar within a gene category represents an independent experiment.

In *Ae.* *aegypti* and a 28-fold upregulation of ‘Duox2’ (Entrez Gene ID 5569376) was observed [11], which was described as an additional ROS-producing enzyme. However, a BLAST comparison and conserved domain analysis of the ‘Duox2’ protein sequence demonstrated that the gene contains neither a peroxidase nor other domains which characterise Duox enzymes [35]. It is instead a DuoxA protein, sharing 68% amino acid identity with the *D. melanogaster* moladietz gene encoding Numb Interacting Protein (NIP), a maturation factor that is essential for the effective functioning of Duox and is thought to aid its transport to the plasma membrane [36,37]. There was no significant increase in transcription of either Duox or DuoxA in *Wolbachia*-infected *Ae.* *albopictus* (Figure 5). There was also no consistent significant change in any antioxidants assayed (Figure 6).

## 4. Discussion

No significant elevation of ROS levels or differential regulation of any of the innate immune pathway regulator genes or of antioxidant/ROS-modulating genes was demonstrated in *Ae.* *albopictus*, except that in cell lines STAT was sometimes upregulated. Overall, these results suggest that the hypothesis of ROS-induced Toll pathway activation is responsible for DENV inhibition in *Ae.* *aegypti* [11] does not apply in *Ae.* *albopictus*. In turn, this suggests that immune upregulation and priming may not be the predominant causal factor of *Wolbachia*-induced DENV inhibition. There was no evidence of immune priming in the presence of *Wolbachia* in the natural host of *w*Mel and *w*MelPop, *Drosophila melangaster*, despite these strain imparting resistance to pathogenic viruses of *Drosophila*, suggesting that immune activation is not a mechanism of viral inhibition common to all species [38,39,40,41]. Immune activation may be a major contributor to *Wolbachia*-mediated DENV transmission blocking in *Ae.* *aegypti* but it is likely that the importance of immune pathways differs across different *Wolbachia*-host-virus interactions.

Increased ROS levels were previously reported by flow cytometry and microscopy in naturally infected and antibiotic-cured *Ae. albopictus* cell lines [28]. Both assays utilised 2',7'-dichlorodihydrofluorescein diacetate (H_2_DCFDA), which is non-specific and will also detect some RNS [42,43]. It is thought to require a transition metal catalyst such as free iron, therefore the localisation of the probe with *Wolbachia*-containing vacuoles reported [28] could be affected by cellular iron distribution and specifically the known sequestration of iron in insect vacuoles [44]. To avoid non-specificity and confounding factors, a H_2_O_2_-specific assay for reduced superoxide was used here, following consideration of alternative assays [42]. The higher variability of results between each independent experiment for the cell lines compared to the adult mosquito assays may be attributed to the heterogeneity of the Aa23 cell line, which contains different cell types; control of culture period and conditions, use of cells in the growth phase at 70%–80% confluency and frequent validation of *Wolbachia* infection via PCR and qPCR were employed to mitigate this inherent variability as far as possible.

The lack of immune activation in *Ae.* *albopictus* also suggests that there has been a co-evolved immune adaptation and potential attenuation of immune responses to *Wolbachia* in this host that has had a long natural history of *Wolbachia* infection. This is in contrast to the chronic immune upregulation that occurs in *Ae. aegypti* when transinfections of *Wolbachia* are made into this naïve host [2,3,10,11]. A potential role of ROS and innate immunity in controlling *Wolbachia* proliferation/infection density is also possible; it is known that innate immunity can play a role in controlling the Rickettsiales *Anaplasma phagocytophilum* and *Ehrlichia chaffeensis* [45,46] and endosymbionts from other phyla have been shown to be regulated by AMPs [47].

The primary causes of virus inhibition in species which do not display immune activation remains open. Some alternative hypotheses have already been discounted, for example siRNA-mediated RNA-interference (RNAi) was not implicated in *D. melanogaster* *Wolbachia*-induced viral immunity [48]. It appears that any antiviral effect is *Wolbachia* density dependent, as inferred from the greater DENV resistance seen in *w*MelPop compared to *w*Mel-infected *Ae.* *aegypti* and *Ae.* *albopictus* cell lines [26] and strengthened by later results showing that *w*AlbB, which does not induce DENV resistance in its low density natural infection of *Ae.* *albopictus*, can do so in a density dependent manner in *Ae.* *albopictus* cell lines [27]. A linear positive correlation between *Wolbachia* density and Defensin D (DEFD) transcription was observed [27]. However, given the lack of evidence for increased AMP production [5] and immune pathway signalling in *Ae.* *albopictus* presented here, alternative hypotheses which are also compatible with density dependence must be pursued. These include *Wolbachia*-mediated modulation of resources such as lipids required by arboviruses for their replication; indeed an effect of cholesterol levels on the inhibition phenotype has already been demonstrated in *Drosophila* [49].
